# Immune checkpoint pathways in glioblastoma: a diverse and evolving landscape

**DOI:** 10.3389/fimmu.2024.1424396

**Published:** 2024-09-13

**Authors:** Julio F. Inocencio, Stefan Mitrasinovic, Mohammad Asad, Ian F. Parney, Xingxing Zang, Benjamin T. Himes

**Affiliations:** ^1^ Department of Neurological Surgery, Montefiore Medical Center/Albert Einstein College of Medicine, Bronx, NY, United States; ^2^ Department of Neurologic Surgery, Mayo Clinic, Rochester, MN, United States; ^3^ Department of Immunology, Mayo Clinic, Rochester, MN, United States; ^4^ Department of Microbiology and Immunology, Albert Einstein College of Medicine, Bronx, NY, United States; ^5^ Department of Oncology, Albert Einstein College of Medicine, Bronx, NY, United States

**Keywords:** immune checkpoints, glioblastoma, immune microenvironment, tumor immunosuppression, immunotherapy

## Abstract

Immune checkpoint (IC) inhibition in glioblastoma (GBM) has not shown promising results in the last decade compared to other solid tumors. Several factors contributing to the lack of immunotherapy response include the profound immunosuppressive nature of GBM, highly redundant signaling pathways underlying immune checkpoints, and the negative immunogenic impact of current standard of care on the tumor microenvironment. In this review, we will discuss various ICs in the context of GBM, their interplay with the tumor immune microenvironment, relevant pre-clinical and clinical studies, and the impact of current treatment modalities on GBM IC blockade therapy. Understanding the molecular mechanisms that drive ICs, and how they contribute to an immunosuppressive tumor microenvironment is critical in advancing IC inhibition therapy in GBM. Furthermore, revisiting current treatment modalities and their impact on the immune landscape is instrumental in designing future combinatorial therapies that may overcome treatment resistance.

## Introduction

The treatment paradigm for Glioblastoma (GBM) has not changed substantially over the past several decades. The standard of care remains based on the Stupp protocol dating back to 2005, which involves safe maximal resection followed by adjuvant chemoradiation therapy (1). Despite numerous advances including the addition of anti-VEGF therapies and tumor-treating fields, the prognosis has remained grossly unchanged with a median survival of 15 to 20 months (2). Due to the inherent heterogeneity of GBM, molecular targeted therapy including inhibitors of EGFRvIII (3), phosphoinositide 3-kinase (PI3K)/protein kinase B (AKT) (NCT00595954), and mammalian target of rapamycin (mTOR)(NCT00515086 and NCT00016328) have failed to produce any survival benefit over standard treatment (4).

The discovery of immune checkpoint (IC) inhibitors in the treatment of other solid tumors such as anti-PD-1 and anti-CTLA-4 for non-small cell lung cancer and melanoma has drastically changed therapeutic management in those diseases achieving as high as 20-30% 5-year survival rates (5, 6). ICs are surface molecules that serve as negative modulators by providing inhibitory signals that prevent immune cell attack and lead to overall self-tolerance. Tumor cells also express these molecules to evade the immune response and promote tumorigenesis. The investigation of ICs and their blockade has therefore been an active area of research in anticancer therapy. Unfortunately, while promising results have been shown in other tumors, current trials of IC blockade (ICB) in GBM have failed to replicate similar positive results (**Table 1**) (7, 8).

**Table 1:** Select clinical trials involving checkpoint blockade therapy in GBM.

	TRIAL	GBM TYPE	PHASE	DRUG	DESIGN	RESULTS
PD-1	NCT 02617589 Checkmate 498	nGBM	3	Nivolumab	n=560 Arm 1: TMZ + RT + Nivolumab Arm 2: TMZ + RT	Overall Survival: No significant difference between arms Arm 1: 13.4 months Arm 2: 14.88 months
	NCT 05235737	nGBM	4	Pembrolizumab	n=36 Arm 1: Neoadjuvant & Adjuvant Pembrolizumab Arm 2: Neoadjuvant Pembrolizumab Arm 3: Stupp protocol only	Not Available
	NCT 03899857	nGBM	2	Pembrolizumab	n=56 (Single Group Assignment) Pembrolizumab + Stupp Protocol	Not Available
	Ivy Brain Foundation Consortium	rGBM	2	Pembrolizumab	n=35 Arm 1: Neoadjuvant & Adjuvant Pembrolizumab Arm 2: Adjuvant Pembrolizumab	Overall Survival: Arm 1: 13.7 months Arm 2: 7.5 months
	NCT 02852655	rGBM	1	Pembrolizumab	n=25 Arm 1: Neoadjuvant & Adjuvant Pembrolizumab Arm 2: Adjuvant Pembrolizumab	Submitted, not available
	NCT 03661723	rGBM	2	Pembrolizumab	n=60 Arm 1: Pembrolizumab + re-irradiation Arm 2: Pembrolizumab + re-irradiation + Bevacizumab	Overall Survival: Arm 1: 11.8 months Arm 2: 8.6 months
	NCT 02337686	rGBM	2	Pembrolizumab	n=18 (Single Group Assignment) Neoadjuvant and Adjuvant Pembrolizumab	Overall Survival: 20 months
	NCT 023337491	rGBM	2	Pembrolizumab Bevacizumab	n=80 Arm 1: Pembrolizumab + Bevacizumab Arm 2: Pembrolizumab	Overall Survival Arm 1: 8.8 months Arm 2: 10.3 months
	NCT 02017717 Checkmate 143 (Phase I Cohorts)	nGBM	3	Nivolumab	n=136 Part A Arm 1: Nivolumab + RT + TMZ Arm 2: Nivolumab + RT Part B (unmethylated MGMT promoter only from Part A randomized into the following arms) Arm 3: Nivolumab + RT + TMZ Arm 4: Nivolumab + RT Cohort 2 Arm 5: Nivolumab Arm 6: Bevacizumab	Overall Survival: MGMT methylated group showed 2x longer OS *vs*. MGMT unmethylated group with Nivolumab + RT + TMZ treatment Part A • Arm 1: 22.08 months • Arm 2: 14.41 months Part B • Arm 3: 14.75 months • Arm 4: 13.96 months Cohort 2 • Arm 5: 9.77 months • Arm 6: 10.05 months
	NCT 02667587 Checkmate 548	nGBM	3	Nivolumab	N=716 Arm 1: Nivolumab + RT + TMZ Arm 2: Placebo + RT + TMZ	Overall Survival Arm 1: 31.34 months Arm 2: 32.99 months
PD-L1	NCT 02866747	rGBM	1,2	Durvalumab	n=108 Arm 1: Hypofractionated RT Arm 2: Hypofractionated RT + Durvalumab	Not Available
	NCT 02336165	nGBM rGBM	2	Durvalumab Bevacizumab	n=159 Cohort A (nGBM): Durvalumab Cohort B (rGBM/Bevacizumab naïve) Arm 1: Durvalumab Arm 2: Durvalumab + Bevacizumab (high dose) Arm 3: Durvalumab + Bevacizumab (low dose) Cohort C (rGBM/Bevacizumab Refractory): Durvalumab + Bevacizumab	Overall Survival: Cohort A: 64.8 months Cohort B: • Arm 1: 39.4 months • Arm 2: 37.3 months • Arm 3: 39.7 months Cohort C: 19.3 months
	NCT 03047473	nGBM	2	Avelumab	n=30 (Single Group Assignment) Avelumab + Standard of Care	No preliminary survival data available iRANO Criteria at 1 year Complete Response: 4 Partial Response: 2 Stable Disease: 3 Disease Progression: 19 Lost to follow-up: 2
	NCT 05423210	nGBM	1	Atezolizumab	n=12 (Single Group Assignment) Atezolizumab + Hypofractionated Radiation	Not Available
CTLA-4	NCT 02017717 Checkmate 143 (Phase I Cohorts)	nGBM	3	Ipilimumab Nivolumab	n=40 Arm 1: Nivolumab + Ipilimumab (high dose) Arm 2: Nivolumab + Ipilimumab (low dose)	No survival data available Nivolumab alone was tolerated better *vs*. combined therapy with higher adverse events in high dose ipilimumab group
	NCT 04817254	nGBM	2	Ipilimumab Nivolumab	n=58 Arm 1: Nivolumab + Ipilimumab (high dose) Arm 2: Nivolumab + Ipilimumab (low dose	Not Available
	NCT 04396860	nGBM	2,3	Ipilimumab Nivolumab	n=485 Arm 1: Standard of Care +/- Optune (TTF) Arm 2: Nivolumab + Ipilimumab + RT	Not Available
	NCT 02794883	rGBM	2	Tremelimumab Durvalumab	n=36 Arm 1: Durvalumab Arm 2: Durvalumab + Tremelimumab Arm 3: Tremelimumab	Overall Survival: Arm 1: 11.71 months Arm 2: 7.7 months Arm 3: 7.2 months
	NCT 04606316	rGBM	1	Ipilimumab Nivolumab	n=63 Arm 1: Nivolumab + Ipilimumab (pre & post surgery) Arm 2: Nivolumab (pre & post surgery) Arm 3: Nivolumab + Ipilimumab (post surgery)	Not Available
LAG-3	NCT 02658981	rGBM	1	BMS-986016 (anti-LAG-3) Urelumab (anti-CD137) Nivolumab	n=63 Arm 1: BMS-986016 Arm 2: Urelumab Arm 3: BMS-986016 + Nivolumab Arm 4: Urelumab + Nivolumab	Not Available
	NCT 03493932	rGBM	1	BMS-986016 Nivolumab	n=21 (single group assignment) BMS-986016 + Nivolumab	Not Available
B7-H3	NCT 04077866	rGBM	1,2	B7-H3 CAR-T	n=40 Arm 1: TMZ only Arm 2: TMZ + B7-H3 CAR-T	Not Available
	NCT 05241392	rGBM	1	B7-H3 CAR-T	n=30 (single group assignment) B7-H3 CAR-T	Not Available
	NCT 05366179	rGBM	1	B7-H3 CAR-T	n=36 (single group assignment) B7-H3 CAR-T	Not Available
	NCT 05835687	(pediatric <21 years old) Primary CNS Tumors including GBM	1	B7-H3 CAR-T	n=36 (single group assignment) B7-H3 CAR-T	Not Available
TIGIT	NCT 04656535	rGBM	1	Ab154 IgG1 (anti-TIGIT) Ab122 IgG4 (anti-PD-1)	n=40 Arm 1: Ab154 (post surgery) Arm 2: Ab122 (post surgery) Arm 3: Ab154 + Ab122 (post surgery) Arm 4: Placebo (post surgery)	Not Available
TIM-3	NCT 03961971	rGBM	1	MBG453 Spartalizumab	n=16 (single group assignment) MBG453 + Spartalizumab	Not Available

In this article, we will discuss the unique immune landscape that shapes the GBM microenvironment and review the diverse IC molecules that have been identified and are currently being investigated in GBM, along with some of the pitfalls of ICB.

## Immunosuppression in GBM

**Figure 1:**
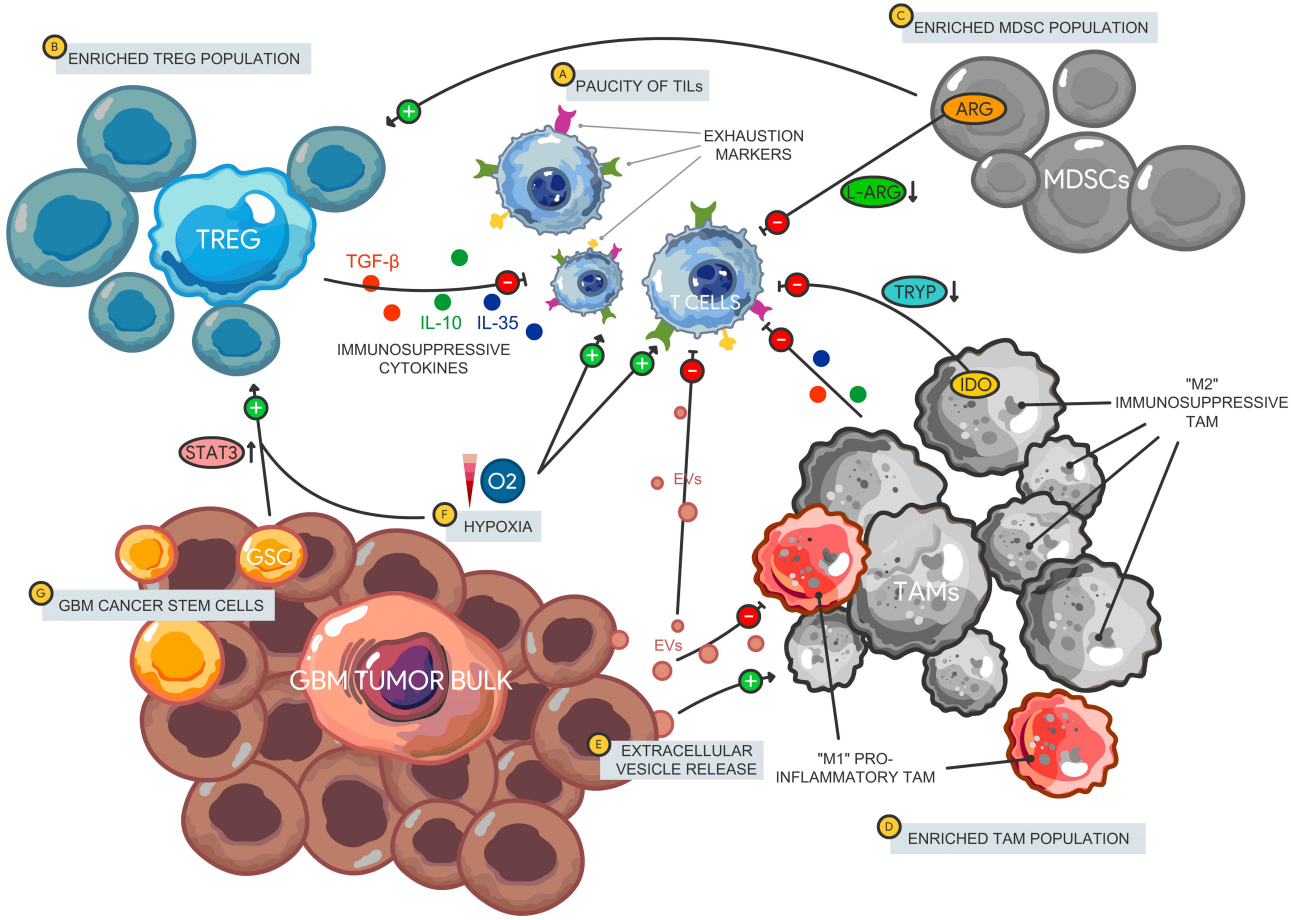
Immune microenvironment in glioblastoma. The GBM immune landscape is characterized by a **(A)** paucity of tumor infiltrating lymphocytes that express multiple immune checkpoints and exhaustion markers leading to an impaired effector cell function and decreased tumor cell killing. There is also enrichment of immunosuppressive cellular subsets including Tregs, MDSCs, and TAMs. **(B)** Tregs release immunosuppressive cytokines including IL-10, IL-35, and TGF-β that impair T cell activation. Furthermore, **(C)** MDSCs promote Treg proliferation and function, while impairing T cell signaling via amino acid depletion (L-arginine) leading to reduced T cell proliferation. **(D)** TAMS comprise most of the immune cell tumor bulk with a larger proportion polarized towards an immunosuppressive phenotype (classically designate “M2”). TAMs impair T cells through tryptophan depletion via IDO expression as well as the release of immunosuppressive cytokines. **(E)** GBM-derived EVs carry bioactive molecules that promote immunosuppressive TAMs and limit the pro-inflammatory subtype. EVs also carry immunosuppressive cargo such as PD-L1 that can disrupt T cell activation and proliferation. **(F)** The hypoxic environment in GBM can also induce gene expression of ICs on lymphocytes through HIF-1α, promote MDSC function via NO production leading to IL-2 signaling inhibition and maintenance of the GSC population. **(G)** GSCs support Treg proliferation via STAT3 signaling and support the TAM population. Tregs, Regulatory T cells; MDSCs, Myeloid-derived suppressor cells; TAM, Tumor-associated macrophages; IDO, indoleamine 2,3-dioxygenase 1; EVs, Extracellular vesicles; HIF-1α, Hypoxia-inducible factor 1 alpha; NO, Nitric oxide.

The various mechanisms of GBM immunosuppression have been extensively studied (9–11) and serve as major barriers to therapeutic access in the realm of ICB. Here, we will focus on the immune cell subsets that contribute to the overall immunosuppressive landscape in GBM that negatively impact ICB efficacy (**Figure 1**). GBM has a distinct immune microenvironment characterized by an overall paucity of lymphocytes (12) with an abundance of other immunosuppressive cell subsets including regulatory T cells (Tregs) (13), tumor-associated macrophages (TAMs), and myeloid-derived suppressor cells (MDSCs) (14). TAMs, which are comprised of bone-marrow derived monocytes/macrophages and resident microglia, constitute the main bulk of immune cells in GBM (15). In stark contrast, CD4+/CD8+ cells only make up to 2% (12) of GBM infiltrating immune cells and a majority of which express exhaustion markers that signal anergy and dysfunction (16). The GBM TME is also characterized by immunosuppressive cytokines including IL-1, TGF-β, and IL-10, as well as factors (CSF-1 and Arginase I) that impair effector T cell function and maintain tumorigenic cellular populations (Tregs, immunosuppressive TAMs, and MDSCs) that ultimately contribute to immune escape (17). In addition, GSCs contribute to upregulating signaling pathways (STAT3) that promote Tregs and block macrophage proliferation (18), while the hypoxic environment enhances these effects via similar signaling pathways (19). Given that the clinically available ICBs primarily target the lymphocytic cellular compartment, it is not surprising that most trials have failed to show a survival benefit compared to standard of care.

TAMs are a heterogeneous population polarized towards various phenotypes including early undifferentiated, inflammatory, or immunosuppressive and reparative. While historically categorized under either a pro-inflammatory (M1) or immunosuppressive (M2) phenotype, these designations were based on *in vitro* experiments that do not accurately represent macrophages in their native environment. They also do not account for the myriad cytokine profiles that may have specific function in different conditions. It is now acknowledged that TAMs exist on a spectrum, expressing a wide range of surface markers that span the spectrum. In the GBM TME, studies have shown that TAMs are generally polarized towards an immunosuppressive phenotype and secrete anti-inflammatory cytokines such as TGF-β and dampen T cell proliferation, activation and cytotoxicity (20–23). These immunosuppressive TAMs are also enriched in hypoxic and necrotic regions of the tumor (24) and are sustained by the GBM cancer stem cell (GSC) population (25).

Tregs are a subset of CD3+ lymphocytes characterized by CD4^+^CD25^+^FOXP3^+^ markers and typically secrete immunosuppressive cytokines namely TGF-β, IL-10, and IL-35 (26). They induce anergy and tolerance on effector lymphocytes and play a critical role in preventing autoimmunity (27). Tregs are enriched in infiltrating immune cells in GBM while are virtually undetected in lower grade gliomas, meningiomas or pituitary adenomas (28) making them an attractive target in GBM therapy. Although their presence in the tumor bulk contributes to T cell effector dysfunction, the prognostic relevance of Treg frequency remains unclear. Some studies demonstrate a negative correlation between Treg enrichment and overall survival (29) while others have shown no clear link (30, 31).

MDSCs on the other hand are a mixed cell subset within the myeloid compartment that lack specific surface markers that characterize more terminally differentiated cells such as dendritic cells, macrophages, and monocytes (240, 241). Identification and characterization of this heterogeneous immune cell population has been constantly evolving. Nonetheless, MDSCs are generally broken down into polymorphonuclear-MDSCs (PMN-MDSCs) defined as CD15^+^CD33^+^HLADR^-^, monocytic (M-MDSCs) defined as CD14^+^CD33^+^HLADR^-^, and early stage (E-MDSCs) subtypes (32). MDSCs primarily exert their immunosuppressive effects through various mechanisms including amino acid depletion, oxidative stress, and indirect induction of Tregs among others. L-Arginine depletion through production of Arginase by MDSCs limits the half-life of CD3ζ mRNA, a critical signaling component of the T cell receptor, which ultimately leads to diminished T lymphocytes and NK cell proliferation (33). Serum arginase has been shown to be significantly elevated in GBM patients and has been linked to an intracellular MDSC activation marker S100A/9 particularly in the PMN-MDSC subset (34). The hypoxic nature of the GMB TME also contributes to further MDSC immunosuppression through inducible nitric oxidase synthase (iNOS) induction which produces nitric oxide (NO) from L-arginine. NO production by MDSCs inhibits proximal IL-2 signaling pathway (JAK/STAT phosphorylation) in an IFN-γ dependent manner, leading to overall impairment of T cell proliferation (35). Similar to arginase, iNOS expression is also correlated with higher tumor grades in gliomas (36) and therefore has been studied as a possible therapeutic target in preclinical models (37, 38). In these studies, iNOS inhibition showed reversal of immunosuppression of MDSCs on T cell proliferation.

Tumor-derived extracellular vesicles (EVs) have also been implicated in maintaining the immunosuppressive milieu in GBM. EVs are bilipid layer particles of varying sizes that are released from various types of cells and contain bioactive molecules including RNA, DNA, and proteins. Apart from transferring oncogenic cargo to neighboring tumor cells (i.e. EGFRvIII protein and mRNA) (39) that lead to conventional therapeutic resistance, GBM-derived EVs have been demonstrated to block T cell proliferation and activation (40), express certain ICs (i.e. PD-L1) (41), polarize TAMs into an immunosuppressive phenotype (42), and induce MDSCs (43). Preventing the interaction and subsequent uptake of EVs by various immune cells may reverse some of their negative immunomodulatory effects (44) and is another potential therapeutic avenue to augment ICB treatment.

Finally, tumor infiltrating dendritic cells and macrophages also express the enzyme indoleamine 2,3-dioxygenase 1 (IDO), which metabolizes tryptophan into kyneurenines. This suppresses T cell proliferation in two ways. Depletion of an essential amino acid tryptophan inhibits further cell cycle progression while tryptophan catabolites (kynurenine) further regulate T cell activation (45). While IDO is not normally expressed in abundance in normal brain tissue, it is upregulated in both GBM tumor cells as well as specific myeloid cells and is further inducible through various cytokines (46). Specifically, IFN-γ upregulates IDO1 in GBM tumor cells and tumor-derived extracellular vesicles (EVs). These IDO1^+^ EVs derived from IFN-γ exposed GBM cells lead to higher induction of MDSCs from monocytes and cause more T cell inhibition compared to EVs derived from IFN-γ naïve GBM cells that have lower IDO1 expression (43). Unsurprisingly, higher IDO levels correlate with overall poorer prognosis in GBM patients and increased recruitment of both Tregs and MDSCs (47, 48). Moreover, The ECHO-301 trial was the first phase 3 trial investigating the effect of anti-IDO and anti-PD1 therapy in melanoma; however, the trial itself had significant limitations and showed no survival benefit (49, 50). There are currently several trials in GBM exploring IDO inhibition alongside Temozolomide, bevacizumab, and anti-PD1 therapy although results are not available currently (NCT02052648 and NCT02502708).

## Immune checkpoints in GBM

### PD-1/PD-L1

Programmed cell death protein-1 (PD-1 or CD279), encoded by the PDCD1 gene, is a receptor belonging to the immunoglobulin superfamily found primarily on the surface of immature T lymphocytes, differentiated T and B cells as well as myeloid cells. It was first discovered in 1992 through subtractive hybridization comparing resting and apoptotic hematopoietic progenitor cells (51) and eventually mapped to chromosome 2q37.3 (52). It is composed of a single IgV extracellular domain, a transmembrane domain, and a cytoplasmic tail composed of two tyrosine residues (1) a tyrosine inhibitory domain (ITIM) and (2) a tyrosine-based switch motif (ITSM). While both cytoplasmic domains have been theorized to bind their respective tyrosine phosphatases, direct interaction has not been demonstrated. Initial studies show that defects in the ITSM and not the ITIM domain were sufficient to abrogate PD-1 signaling (53). However, more recent evidence demonstrates that a specific mutation in the ITIM domain at position Y248 can impair PD-1 mediated IL-2 production (54).

PD-1 plays a vital role in immune homeostasis and prevents unchecked autoimmunity and inflammatory reaction cascades (55). In GBM, however, the elevated expression of the PD-1 ligand (PD-L1) by tumor cells, antigen presenting cells (APCs), and other cell populations including endothelial cells, pericytes and fibroblasts leads to T cell impairment allowing for immune surveillance evasion and escape (56). In fact, PD-L1 expression correlates positively with higher glioma grade, IDH-wildtype status (57), and the mesenchymal subtype (58).

**Figure 2:**
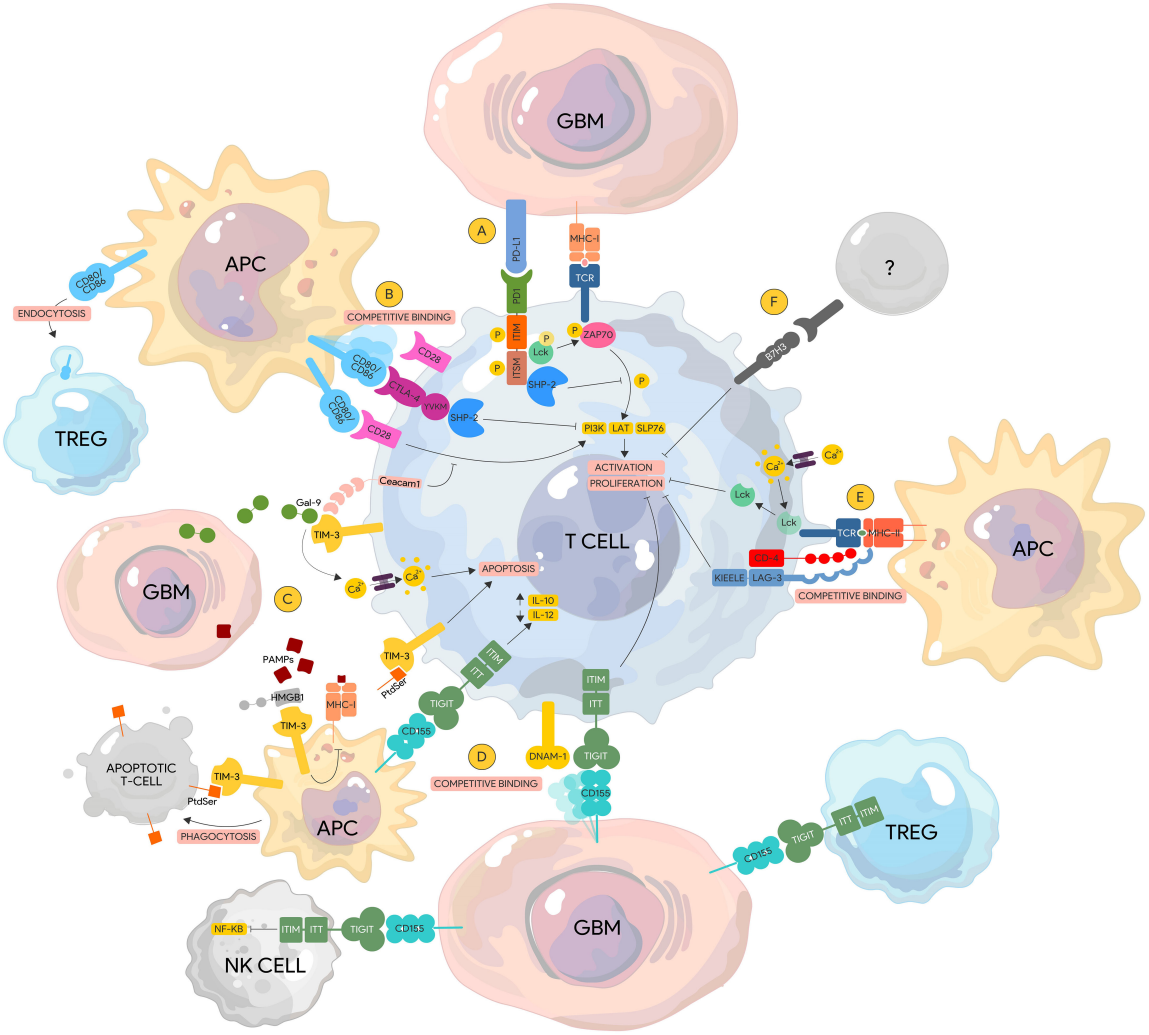
Immune checkpoint pathways in glioblastoma. **(A)** PD-1. PD-1/PD-L1 binding leads to the recruitment and activation of the SHP-2 phosphatase by the ITIM and ITSM domains, which de-phosphorylates ZAP70 and downregulates subsequent downstream proteins (i.e. PI3K, LAT, SLP76) resulting in lymphocyte activation and proliferation. ZAP70 phosphorylation by Lck is also inhibited by the ITSM domain of PD-1 impairing downstream TCR signaling. **(B)** CTLA-4. CTLA-4 is a homolog of CD28 and has a higher affinity to their common ligand CD80/86 thereby displacing CD28. Competitive binding of CTLA-4 to CD80/86 recruits SHP-2 to its YVKM motif leading to the downregulation of similar signaling pathways that lead to T cell activation. **(C)** TIM-3. Gal-9 binding to TIM-3 results in calcium influx that leads to lymphocyte apoptosis. Similarly, PtdSer from apoptotic cells can bind to TIM-3 expressed on APCs leading to phagocytosis. In contrast, binding of PtdSer to TIM-3 on lymphocytes inducing apoptotic signals instead. HMGB1 competitively binds TIM-3 displacing PAMPs from apoptotic tumor cells inhibiting the processing of these PAMPs and their expression via MHC-I leading to tumor surveillance escape. Ceacam1 and TIM-3 are co-expressed on lymphocytes and function to regulate co-stimulatory signals from the CD28-CD80/86 complex leading to immunosuppression. **(D)** TIGIT. TIGIT competitively binds CD155 displacing the stimulatory protein DNAM-1 leading to lymphocyte exhaustion and anergy. Binding of TIGIT expressed on other immune cells to CD155 on tumor cells can lead to reduced NK Cell cytotoxicity via downregulation of the NF-KB pathway and increased immunosuppressive capacity of TREGs. **(E)** LAG-3. LAG-3 has a higher affinity to the TCR/MHC-II complex than CD-4. Binding of LAG-3 to the TCR/MHC-II complex leads to lymphocyte impairment via the cytoplasmic KIEELE motif. In the absence of MHC-II binding, acidification within the LAG-3 immunological synapse via calcium influx results N the dissociation of Lck from the TCR complex, preventing its ability to phosphorylation downstream activator proteins. **(F)** B7-H3. The ligands of B7-H3 are currently unknown. Activation of B7-H3 leads to impairment of lymphocyte activation and proliferation. DNAM-1, DNAX accessory molecule-1; Gal-9, Galectin-9; TCR, T-cell receptor; Gal-9, Galectin-9; PtdSer, Phosphatidylserine; MHC-I TIM-3, T cell immunoglobulin and mucin-domain containing-3; TIGIT, T cell immunoreceptor with Ig and ITIM domains; Ceacam1, Carcinoembryonic antigen-related cell adhesion molecule 1; HMGB1, High mobility group box 1 protein.

PD-1/PD-L1 axis has multiple regulatory mechanisms. Direct binding of PD-1 to its ligands (PD-L1 and PD-L2) leads to recruitment of protein tyrosine phosphatases SHP-1 and SHP2 by the cytoplasmic domains ITIM and ITSM, respectively (**Figure 2**). This interaction in turn dephosphorylates downstream effector molecules such as Zap70/CD3ζ in T cells, and Syk/PI3K in B cells (59), which results in the blockade of the TCR (T cell receptor) signaling pathway. These effector molecules are proximal activators of the MAP/ERK/JNK signaling pathways responsible for cytokine production, proliferation, activation, and survival. Direct binding of PD-1 to its canonical ligands therefore leads to immune cell exhaustion, anergy, and eventual apoptosis.

Multiple signaling pathways affect PD-L1 expression. PTEN, a tumor suppressor gene, is a strong inhibitor of the tumorigenic PI3K-Akt-MTOR pathway that drives cell proliferation, invasion, and survival (60). In PTEN mutated GBM, uninhibited Akt signaling leads to recruitment of the PD-L1 transcript to active polysomes for translation, leading to increased PD-L1 protein expression, which can be reversed with rapamycin, an mTOR inhibitor (61). Interestingly, PD-L1 itself blocks casein kinase 2 (CK2), which phosphorylates and stabilizes PTEN, leading to a cyclical increase in PD-L1 expression (62). It is unsurprising that PTEN loss is correlated with impaired tumor site infiltration and thus resistance to anti-PD-1/PD-L1 therapy (63).

Hypoxia, one of the hallmarks of GBM, can also induce PD-L1 expression through upregulation of the transcription factor hypoxia inducible factor-1α (HIF-1α), which binds to the PD-L1 promoter (64). HIF1- α, however, is not solely regulated under oxygen-tension. Loss of PTEN stabilizes HIF1- α via Akt signaling (65), which illustrates a redundant mechanism via the PTEN-PI3K-Akt-mTOR pathway in controlling PD-L1 expression. Despite HIF1-α being an attractive target in GBM, current available inhibitors have non-specific off-targets (66). The nitrone compound OKN-007, which targets the TGFB1 pathway as well as VEGFR2a and HIF-1α, is currently being investigated in a handful of clinical trials in newly diagnosed and recurrent GBM (NCT01672463, NCT00561374, and NCT04388475). These studies are still in the early phase and no survival data is available.

Several other signaling pathways affect PD-L1 expression in GBM including the interferon gamma (IFN-*γ*)/JAK/STAT (67) and the Epidermal Growth Factor Receptor (EGFR)-MAPK/ERK axes (68). While IFN-*γ* is classically a pro-inflammatory cytokine and activator of macrophages and major histocompatibility complex class (MHC) II, it has been demonstrated to increase PD-L1 expression via binding of the IRF1 transcription factor to the PD-L1 promoter (69). The EGFR/MAPK/ERK pathway on the other hand works in conjunction with CSN6, part of an eight-protein complex COP9 signalosome family (CSN), to stabilize PD-L1 post-transcriptionally and prevent its degradation (68).

Although anti-PD-1/PD-L1 therapy has shown promise in several tumors, blockade of this axis in GBM has not resulted in any significant survival advantage compared to standard care (**Table 1**). In newly diagnosed MGMT-unmethylated GBM, nivolumab (anti-PD-1) monotherapy showed no increased survival benefit when compared to TMZ after radiation and surgical resection (median overall survival was 14.9 months in the TMZ arm compared to 13.4 months in the nivolumab arm) (NCT02617589). The addition of nivolumab to TMZ when compared to TMZ alone also showed no survival benefit in newly diagnosed GBM (NCT02667587). In a large, randomized study (Checkmate 143) comparing nivolumab monotherapy versus nivolumab with bevacizumab, an anti-VEGF treatment commonly used for relapsed disease, no significant difference in overall survival was seen in recurrent GBM (9.7 months in Nivolumab monotherapy *vs*. 10.05 months in combination therapy) (70). The lack of anti-PD1 therapeutic response in GBM is multifold. While PD-L1 expression quantified as the tumor proportion score (TPS) or combined proportion score (CPS) is prognostic in other tumors (71, 72). GBM has an immensely varied PD-L1 expression ranging from 6% to approximately 80% and does not seem to predict clinical response (17, 73). In addition, high tumor mutational burden (TMB) contributes to neoantigen expression and correlates with immunotherapy response in many cancers (74–76). GBM, however, generally exhibits an intermediate TMB (77) with no clear correlation between higher TMB and immunotherapy response (78). In fact, certain studies have shown the opposite trend with lower TMB showing possible favorable immunotherapy response (79). In GBMs with relatively elevated TMB, prognosis is poorer possibly due to the mutations occurring at genes required for chemotherapeutic response (i.e. mismatch repair proteins) (80).

Zhao and colleagues investigated genetic characteristics that differentiated between anti-PD1 responders and non-responders (81). They found that PTEN and MAPK pathway mutations along with activation of PI3K-Akt signaling were higher in non-responders, which is not surprising given the role of PTEN in PD-L1 stabilization as discussed previously. PTEN wildtype tumors also showed higher levels of T lymphocytic infiltration post-immunotherapy compared to matched pre-therapy samples, further suggesting a key role of PTEN in shaping the immune microenvironment in GBM. Interestingly, no correlation between GBM subtype and clinical response was found, despite PD-L1 levels correlating more with the mesenchymal subtype (82). Exposure to anti-PD1 has also been shown to result in the upregulation of other immune checkpoints in other tumor models. In a mouse model of NSCLC, Koyama and colleagues found increased expression of alternate immune checkpoints particularly TIM-3 during disease progression post anti-PD-1 therapy (83). Upregulation of these immune checkpoints was found only on tumor-infiltrating lymphocytes but not on peripheral cells (i.e. lymph nodes, peripheral blood, or spleen) and only at tumor relapse suggesting a post-treatment resistance pattern. Unfortunately, no increased survival in their *in vivo* model was seen with a combinatorial anti-PD-1 and anti-TIM-3 therapy, suggesting other compensatory mechanisms for persistent immunosuppression.

Several pre-clinical studies have investigated multimodal therapies combining immune checkpoint inhibitors with specific molecular targets on immunosuppressive cells to circumvent therapeutic resistance. CSF-1 Receptor (CSF1R) is critical in macrophage differentiation and survival, and inhibition of this pathway in GBM mouse models has shown glioma regression through polarization of tumor associated macrophages (TAM) towards an inflammatory phenotype (84). Combinatorial therapy of anti-CSF1R and anti-PD1 demonstrated prolonged survival in GBM mouse models compared to monotherapy (85). Interestingly, this effect was only seen if both drugs were administered simultaneously or if CSF1R blockade preceded PD-1 blockade. Similarly, inhibiting the tumor-released immunomodulatory chemokine CXCR4 in combination with anti-PD1 demonstrated increased overall survival in GBM mouse models along with a decrease in regulatory FOXP3+ T cells and an elevation in inflammatory cytokine levels (IFN-*γ*/TNF-a) (86). These findings further support the principle that mechanisms underlying resistance against ICBs are tied to the immunosuppressive tumor environment and immunomodulation may be required as an adjunct.

Several clinical trials are currently underway investigating anti-PD1 in combination with other immune checkpoint inhibitors (NCT03961971, NCT03233152, and NCT02658981). However, while pre-clinical GBM models of combinatorial ICI therapy have demonstrated some survival benefit (87), the immune landscape in these mouse models may not be representative of the tumor microenvironment in humans ultimately leading to therapeutic failure.

### CTLA-4

The inhibitory receptor cytotoxic T lymphocyte protein 4 (CTLA-4) was first identified in 1987 via subtractive DNA screening of cytotoxic T cells (88). Like PD-1, it functions to counteract and regulate T cell proliferation and cytokine production that leads to anergy. *In vitro* experiments show CTLA-4 blockade results in uncontrolled T cell proliferation while CTLA-4 deficient mouse models develop fatal lymphoproliferative disease early in life (89, 90). CTLA-4 shares structural homology with CD28, a co-stimulatory protein that provides secondary signals required for T cell activation. Both CTLA-4 and CD28 bind to the same ligands on antigen-presenting cells namely CD80 and CD86; however, CTLA-4 has been demonstrated to have a higher affinity to both ligands compared to CD28 (91).

CTLA-4 is expressed in activated T cells and is barely detectable on the surface of resting lymphocytes (92). The exception to this is FOXP3+ regulatory T cells where CTLA-4 is constitutively expressed (93). Upon T-cell activation, IL-2 production is increased which upregulates CTLA-4 expression and its translocation from the golgi apparatus to the cellular membrane (94). CTLA-4 can be detected on the cell surface as early as 24 hours, peaking at 48 hours, and progressively declining by 72 hours. In contrast, CD28 is expressed on both T cell subsets even at resting states (95). This expression pattern may have major implications on administration timing of CTLA-4 blocking antibodies.

CTLA-4 exerts its regulatory role in two major ways either via cell-intrinsic (directly affecting the cell that expresses CTLA-4) or cell-extrinsic (affecting other cellular compartments that lead to T cell inactivation) mechanisms. CTLA-4 can abrogate lymphocyte activation by directly outcompeting CD28 binding to CD80/86 due to its higher affinity to these ligands. Its cytoplasmic tail of CTLA-4 also contains a YVKM motif that binds to PI3K and recruits phosphatases (SHP-2 and PP2A) that can disrupt downstream TCR signaling (96) similar to PD-1. CTLA-4 is also able to significantly reduce the contact time between APCs and T-cells at the immunological synapse, which is required for proper lymphocyte activation (97).

CTLA-4 can also exert negative regulatory effects through cell-extrinsic pathways by influencing other subsets of immune cells. As previously mentioned, CTLA-4 is constitutively expressed in regulatory T cells and is a direct target of the transcription factor FOXP3 (98). Although CTLA-4 is not required for normal Treg development, it plays a critical role in the suppressive function of Tregs and the prevention of autoimmunity (99). Moreover, CTLA-4 expressing Tregs can deplete CD80/86 on dendritic cells via trans-endocytosis rendering them ineffective in priming T cells (100). Furthermore, binding to CD80/86 on APCs causes a signaling cascade that leads to increased IDO expression in APCs, blocking T cell proliferation via tryptophan depletion (101).

The extensive role of CTLA-4 in negatively regulating the immune response and maintaining homeostasis has made it an interesting target for cancer immunotherapy. The anti-tumor effect of CTLA-4 blockade *in vivo* was first demonstrated by James P. Allison and colleagues (102) and has since been demonstrated in various clinical trials to result in durable responses and improved survival in a subset of patients (103, 104).

Despite promising results in certain cancers, there are several challenges surrounding CTLA-4 blockade therapy. Given its critical function in maintaining self-tolerance, the adverse event rate is considerably high with CTLA-4 ICB compared to anti-PD-1/PD-L1, reaching double the rates in some clinical trials (105). In the Checkmate 143 trial (NCT02017717), higher dose ipilimumab in combination with nivolumab showed significantly more adverse events than when a lower dose of ipilimumab was used. Unsurprisingly, the nivolumab arm alone was better tolerated and toxicity is a major hurdle with anti-CTLA-4 treatment. Several studies involving anti-CTLA-4 blockade in GBM are underway, although there is currently limited survival data from these studies so far. A separate trial (NCT02794883) showed that durvalimab (anti-PD-1) was superior to both combinatorial therapy (tremelimumab with durvalimab) or durvalimab monotherapy alone. The adverse event rate was comparable in all three arms suggesting that the addition of CTLA-4 did not contribute significantly to other causes of mortality beyond the primary disease. It is likely that other redundant immunosuppressive pathways beyond both CTLA-4 and PD-1/PD-L1 axes are at work in GBM, which can explain the lack of response with combinatorial therapy. CTLA-4 also functions upstream of PD-1, whereby blockade of T cell proliferation happens upon engagement of the TCR with its ligands in naïve T cells. The CTLA-4 ligands CD80 and CD86 are also more restricted to APCs. In contrast, PD-1 is also expressed in effector T cells while its ligands PD-1/PD-L1 are found in a variety of cells including tumor cells (106). It is possible that the in the setting of GBM, the PD-1/PD-L1 axes has a more significant contribution to immunosuppression that the CTLA-4 axis, which could explain the lack of synergism between the two drugs.

### LAG-3

Lymphocyte activation gene-3 (LAG-3 or CD223), first discovered in 1990, is a transmembrane protein (498 amino acids) expressed primarily on the surface of activated T lymphocytes and NK cells (107) but has also been demonstrated in APCs and certain tumor cell lines (108). Like PD-1, LAG-3 functions to inhibit the T cell receptor (TCR) signaling cascade and regulate activation and proliferation. Similarly, LAG-3 deficiency has thus been implicated in autoimmune conditions such as multiple sclerosis and diabetes (109) although knockout mouse models do not confer a lethal phenotype unless compounded with another IC deficiency such as PD-1 (110, 111). Furthermore, the absence of LAG-3 does not appear to affect normal lymphocyte development further suggesting a regulatory role rather than a survival function (112).

While the structure of LAG-3 has been well defined, the exact mechanism underlying its inhibitory function has not been fully elucidated. It has an extracellular component composed of four domains (D1 to D4), a transmembrane domain, and an intracellular component with a serine phosphorylation site, an EP motif and a KIEELE motif. The KIEELE sequence has been demonstrated to be primarily responsible for the inhibitory effect of LAG-3 as mutants lacking this segment fail to abrogate TCR signaling (113). LAG-3 is embedded in the CD4 locus and shares 20% of its structural homology. It binds at a higher affinity than CD4 to its canonical ligand MHC II (114); however, the inhibitory mechanism of LAG-3 goes beyond competitive binding and displacement of CD4 at the immunological synapse. It is demonstrated that even in the absence of MHC II binding, acidification of the intracellular domain via calcium influx within the vicinity of the TCR causes dissociation of the Lck kinase from the TCR co-receptors. Sequestration of this kinase by the KIEELE motif leads to dephosphorylation and inactivation of downstream effector molecules ZAP-70 and CD3ζ leading to early disruption of the TCR signaling pathway (115).

Indeed, other ligands have been identified such as L-SECtin, Gal-3, FGL-1, a-synPFF and the TCR/CD3 complex directly, although the exact mechanism and downstream effects of ligation to these molecules remain controversial (116). Upon lymphocyte activation, LAG-3 is translocated from the lysosomal storage compartment to the cell membrane surface in a PKC-dependent manner. Sustained activation of T Cells via other extracellular signals (i.e. antigenic exposure) leads to even more upregulation of LAG-3 expression presumably to circumvent over-activation. LAG-3 also exists in a soluble form, which is a result of cleavage at the junction between the D4 and transmembrane domains by two metalloproteinases, ADAM10 and ADAM17. ADAM10 constitutively cleaves LAG-3 into its soluble form while ADAM17 exerts its effects via TCR signaling in a PKC dependent manner (117). This has been suggested as a mechanism to self-regulate the inhibitory function of LAG-3 as mutants of the LAG-3 molecule that do not self-cleave demonstrate a more significant inhibitory effect. Moreover, shRNA targeting ADAM10 demonstrated decreased T cell proliferation (117) presumably allowing unopposed LAG-3 activity. Soluble LAG-3 (sLAG-3), however, retains its high affinity to MHCII and is a powerful adjuvant for antigen presenting cell activation, maturation, and migration to secondary lymph organs to prime naïve T cells (118). Unlike conventional adjuvants that function through the activation of the toll-like receptor signaling pathway, binding of sLAG-3 to MHC II on lipid rafts activates specific downstream signaling pathways that lead to DC activation, which cannot be mimicked by other anti-MHCII antibodies (119). Interestingly, while the presence of detectable sLAG-3 correlates with better prognosis in some tumors such as breast cancer suggesting increased tumor immunity (120), the opposite trend was observed in others (121, 122). It is unclear whether sLAG-3 by itself has its own signaling capacity in the context of tumor or whether it only serves as a biomarker for decreased LAG-3 activity.

LAG-3 also plays a critical role in Treg physiology (123, 124). In cancer, a small population of CD4^+^CD25^hi^FOXP3^+^ Tregs expressing LAG-3 is preferentially expanded in the PBMCs of cancer patients compared to healthy controls as well as within the tumor bulk itself. These CD4^+^CD25^hi^FOXP3^+^LAG-3^+^ T regs produce IL-10 and TGF-β but not IL-2; however, their suppressive effects are dependent on cell contact despite production of immunosuppressive cytokines at least *in vitro* (125). In addition, a small population of induced Tregs marked by CD4^+^CD25^lo^FOXP3^-^ also demonstrates LAG-3 expression and production of IL-10. LAG-3 was shown to be critical for this population to exert T cell suppression as anti-LAG-3 antibodies abrogated such effect both *in vitro* and *in vivo* (123). Finally, LAG-3 can also function in a cell-extrinsic manner by inhibiting dendritic cell maturation through engagement of MHC II. This was shown to be via an ITAM-mediated mechanism involving the SHP1 inhibitory signaling pathway (126).

The varied mechanisms by which LAG-3 exerts a negative regulatory role in the immune system in addition to studies showing synergy with PD-1 make it an attractive target for cancer immunotherapy. After PD-1/PD-L1 and CTLA-4, it is the third immune checkpoint to have been targeted in the clinical setting with a phase 1 trial of relatlimab in 2013 (NCT01968109). In 2022, Opdualag, a combination of nivolumab plus relatlimab, was FDA approved based on the positive results of the RELATIVITY-047 (NCT03470922) trial demonstrating improved progression free survival in advanced melanoma with combination therapy (127). Several other clinical trials involving relatlimab in other tumor types such as colorectal carcinoma, advanced chordoma, acute myeloid leukemia and squamous cell carcinoma are also currently underway (NCT03623854, NCT04080804, NCT04913922, NCT03642067). In a preclinical GBM model, combinatorial therapy of PD1 and LAG-3 blockade showed increased survival over no treatment, but the survival benefit did not reach statistical significance when compared to that of either anti-PD1 or anti LAG-3 therapy alone (87). The investigators also saw a significant survival advantage with earlier administration of anti-PD1 or anti-LAG-3 therapy on day 7 instead of day 10. Not surprisingly, LAG-3 knockout mice treated with anti-PD-1 demonstrated the best overall survival, suggesting that LAG-3 blockade may be efficacious if targeted at an earlier time point. A phase I trial exploring the safety of relatlimab or urelumab (anti-CD137) alone or in combination with nivolumab in recurrent glioblastoma was completed in October 2023. Preliminary results from the study, however, demonstrated no significant increase in OS in the combination therapy arm of anti-LAG-3 and anti-PD1 versus standard of care (NCT02658981). To date, no other clinical trials investigating LAG-3 blockade therapy in glioblastoma are under investigation.

The lack of a similar response in GBM to anti-LAG-3 therapy may be due to several reasons. LAG-3 expression is highly varied in GBM ranging from 10% (128) to 66% (87) of patients depending on the study. However, the percentage of LAG-3+ TILs in the GBM TME is <1% (129), whereas it can reach approximately 5% in melanoma patients. It has been shown that anti-LAG-3 responders typically have >1% LAG-3+ TILs, which could explain a more robust therapeutic effect in melanoma (129). The role of LAG-3 cleavage and soluble LAG-3 is also unclear. In melanoma, pretreatment levels of serum sLAG-3 correlated with anti-PD1 treatment resistance but this effect was not observed with soluble PD1 or PDL1 (130). The mechanism was attributed to specific dysregulation of the CD4^+^ T cell population rather than direct inhibition of CD8^+^ or Tregs. Unsurprisingly, higher levels of ADAM10 correlated with anti-PD1 responsiveness due to increased LAG-3 shedding (131). ADAM10 is among many different metalloproteinases that contribute to multiple signaling pathways that are dysregulated in many tumors (132). In GBM, ADAM10 has been found to affect cell migration and differentiation (133) as well as the NOTCH1 signaling pathway (134), which affects stemness and proliferation. ADAM10 clearly plays a critical role in not only regulating tumor cell intrinsic physiology but also shaping the GBM TME by affecting IC levels and expression as is the case with LAG-3.

### B7-H3

B7H3 or CD276/B7RP-2 is part of the B7 family of proteins that include both PD-L1 and PD-L2. Initially observed to be a costimulatory ligand by enhancing CD4+ and CD8+ T cell induction and IFN-γ production (135), further studies demonstrate a more predominantly negative regulatory function in the adaptive immune system (136, 137). While it shares approximately 20-30% structural homology to other B7 family proteins, it does not share any of the known ligands within the same group (135, 138), and its putative receptor remains elusive (139). It is a type 1 transmembrane protein consisting of an extracellular domain with two identical pairs of IgV and IgC domains, a transmembrane domain, and a short cytoplasmic tail with no known signaling motif (140). Like the other ICs, B7-H3 also exists in a soluble form as a result of MMP cleavage and is detectable in serum (141), although the clinical relevance of its soluble form as a biomarker remains unclear. B7-H3 mRNA is broadly expressed in several tissues at low levels and post-transcriptional levels are tightly controlled even in the setting of immune cell activation. For example, IFN- *γ* has been demonstrated to increase B7-H3 mRNA levels in dendritic cells but the overall surface membrane protein expression remained constant pointing to a possible negative feedback mechanism (136). Nonetheless, the lack of a defined receptor makes characterization of its function and signaling pathway elusive.

B7-H3 has both immunogenic and non-immunogenic roles in cancer. It contributes to excessive proliferation in certain cancers through cell cycle checkpoint dysregulation (142) and is also implicated in increased epithelial to mesenchymal transition, migration, and invasion (143). B7-H3 is also found in high levels in glioblastoma, and was found to be correlated with a poorer prognosis (144), maintenance of the stem cell phenotype, upregulation of tumorigenic signaling pathways (TGF-β and MYC), and increased invasion (145). Decreased B7-H3 expression also correlated with CD8+ T cell infiltration within the TME and increased susceptibility to NK cell killing (146) further suggesting that B7H3 contributes to a “cold” immunogenic microenvironment in GBM. Because of the lack of a defined receptor, developing a blocking antibody specifically against B7-H3 may not guarantee abrogation of its function. Various strategies have been employed to target B7-H3 including a monoclonal antibody with enhanced Fc region binding, antibody-drug conjugates (ADC), and CAR-T cells (147).

Enoblituzumab or MGA271, is a humanized IgG 1κ monoclonal antibody against B7-H3 with an enhanced Fc region binding and was initially developed in 2012 to induce improved antibody-dependent cellular cytotoxicity (148). It entered phase I clinical trials (NCT02982941, NCT01391143, NCT02923180) for various B7-H3 expression solid tumors with acceptable toxicity profiles and evidence of disease stabilization in certain patients. These results led to Phase II trials in both prostate cancer as a neoadjuvant (NCT02923180) and head and neck squamous cell carcinoma (HNSCC) in combination with other ICBs namely retifanlimab (anti-PD-1) and tebotelimab (anti-CTLA-4) (NCT04634825). While preliminary results demonstrated reduced Gleason scores in 50% of prostate cancer patients, the trial for HNSCC was terminated due to a high adverse event rate including tumor hemorrhagic conversion.

ADCs are antibodies conjugated to a drug molecule by a cleavable linker leading to delivery of the drug payload upon internalization of the compound (149). They have been used in certain tumors such as breast cancer and NSCLC targeting HER-2 with demonstrable survival benefit (150, 151). B7-H3 specific ADCs including MGC018 (NCT5293496) and DS-7300 (NCT04145622) are currently under investigation in several solid tumors but not yet in glioblastoma.

In preclinical studies, CAR-T cell therapy targeting B7-H3 has shown potent antineoplastic effect in certain cancers including neuroblastoma, pancreatic cancer, and ovarian cancer (152, 153). In GBM, CAR-T therapy has been used primarily to target EGFRvIII, HER2, and IL-13Ra2 but inherent heterogeneity and poor antigenic expression have been major hurdles in achieving significant therapeutic effect (154). An early preclinical study involving CAR-T Cell therapy targeting B7-H3 showed anti-tumor effect compared to vehicle control treated group with complete remission for up to 2 months (155). The study, however, used implanted GBM cell lines that express B7-H3 in a stable fashion, which limits the effect of tumor heterogeneity. The same group later presented a case report involving a 56-year-old female patient who was treated with B7-H3 targeted CAR-T Cells after recurrence of her GBM 3 weeks post-surgical resection. The patient demonstrated early reduction of tumor size via volumetric MRI measurements but relapsed approximately 50 days after initiation of therapy (156). Like most CAR-T cell regimens, antigenic variability and low expression of the target in surviving tumor cells likely contributed to recurrence. Nonetheless, given the anti-neoplastic effect of targeting B7-H3 in GBM, several phase I trials using CAR-T therapy are currently underway (NCT05241392, NCT04385173, NCT04077866, NCT05366179).

### TIGIT

T cell immunoglobulin and ITIM domain (TIGIT) was first identified in 2009 from a genome wide search of immunomodulatory genes. It is part of the polioviruses receptor family of proteins and contains an IgV domain, a transmembrane domain, and an ITIM and Ig tail-tyrosine (ITT)-like motif (157). TIGIT is a coinhibitory regulatory molecule expressed on activated T cells and highest in regulatory T cells, CD4+CD45RO+ memory T cells, and NK cells subsets (157, 158). It is also present to a smaller extent in naïve T cells, dendritic cells, and TAMs, particularly those polarized towards the immunosuppressive subtype (157, 159).

TIGIT belongs to a family of proteins that include DNAM-1 and CD96 and binds to nectin-like adhesion molecules including CD155, CD112, and CD113. DNAM-1 (CD226) is a costimulatory molecule while CD96 functions as a negative regulatory ligand similar to TIGIT. These three molecules are co-expressed on T cells as well as NK cells and function to fine-tune their activation and cytokine production. TIGIT primarily binds to CD155 and has the highest affinity compared to both DNAM-1 and CD96. CD155, also known as the poliovirus receptor, is expressed in various cell types including monocytes, dendritic cells, endothelial cells and in significant levels in specific tumors such as lung adenocarcinoma, pancreatic cancer, and gliomas (160, 161). It also plays a key role in tumor cell motility, invasion, and migration (162). TIGIT has various mechanisms by which it negatively regulates the immune system. Initial studies supported a cell-extrinsic pathway demonstrating that binding of CD155 on dendritic cells to TIGIT results in a tolerogenic phenotype characterized by increased IL-10 production and decreased IL-12 levels leading to impaired T cell activation (157). TIGIT also competes directly with DNAM-1 by preventing its homodimerization with CD155 thereby abolishing its costimulatory signal. This was apparent when DNAM-1 blockade abrogated the anti-tumor effect of anti-TIGIT and anti-PD1 combinatorial therapy (163). While a study demonstrated that negative effect of TIGIT was specific to CD8+ T cells (163), this was later shown to be via the induction and maintenance of a highly suppressive Treg population (164). The exact mechanism and signaling pathway, however, was not fully elucidated. Nonetheless, a separate study using agonistic antibodies to TIGIT in an APC free system showed decreased T cell activation but showed no effect in TIGIT^-/-^ T cells supporting a cell-intrinsic mechanism as well (165). TIGIT also impairs NK cells directly through recruitment of SHIP-1 phosphatase by the ITIM domain via B-arrestin 2, leading to deactivation of the NF-KB signaling pathway. This results in decreased cytotoxicity and suppression of granule polarization (158, 166). Finally, TIGIT promotes a shift towards the immunosuppressive phenotype in TAMs by increasing nuclear translocation of c-Maf inducing IL-10 transcription (159, 167). A new study reveals that KIR2DL5, a newly identified receptor for CD155, binds to CD155 without competition with TIGIT. Blockade of KIR2DL5, but not of TIGIT, enhances human NK cell function (168).

The multifaceted effect of TIGIT in regulating several immune cell compartments may play a unique role in the GBM TME. TIGIT has been shown to be differentially increased in GBM TILs compared to those in multiple sclerosis (169). While the costimulatory molecule DNAM-1 was co-expressed with TIGIT in GBM TILs, the higher affinity of TIGIT to CD155 as well as the disruption of DNAM-1 homodimerization by TIGIT interaction *in cis* (163) may contribute to a predominantly immunosuppressive effect. Lucca and colleagues also found that PD-1/PD-L1 expression levels were comparable between GBM and multiple sclerosis, suggesting that TIGIT is likely exerting its own unique inhibitory effect in the TME regardless of heavy co-expression with other ICs (170–172).

In preclinical models, TIGIT blockade has shown improved survival, increased CD8+ effector function, and decreased immunosuppressive activity of both Tregs and MDSCs (173, 174). The combination treatment of anti-PD1 and anti-TIGIT has also demonstrated a survival benefit over monotherapy alone in multiple studies including in GBM models (163, 175, 176). In addition to increased IFN-γ production and CD8+ T cell effector function, a decrease in TREG function, frequency of tumor infiltrating DCs, as well as MDSC numbers were also observed, including rescue of T cell proliferation from MDSC suppression (175, 176).

Several clinical studies involving anti-TIGIT therapy and in combination with other ICBs are currently underway in many tumors (NCT02964013, NCT05645692, and NCT05130177). Presently, there is only 1 multicenter clinical trial in early phase 0/1 for recurrent GBM exploring anti-TIGIT (domvanalimab) in combination with anti-PD1 (NCT04656535). The study is actively recruiting and aims to characterize the safety profile of the anti-TIGIT drug AB154 with or without the anti-PD1 drug AB122.

### TIM-3

T cell immunoglobulin and mucin domain containing molecule (TIM-3) was first identified in 2002 as an immunomodulatory transmembrane protein specific to CD4 TH1 helper T cells and CD8+ TC1 cytotoxic T cells (177). Further studies show that it is also expressed on dendritic cells, B cells, macrophages, NK cells, Tregs, memory cells (178, 179) and even in GBM tumor cells.

The structure of TIM-3 consists of a variable ectodomain followed by a mucine-like region, a transmembrane domain, and a cytoplasmic tail containing a tyrosine phosphorylation motif. This motif can associate with Fyn, a src family tyrosine kinase, and the PI3K adapter P85 without the need for ligand binding of the ectodomain (180). This leads to an acute stimulatory effect on T lymphocytes resulting in augmented activation via TCR signaling but can be abrogated with the addition of an agonistic antibody to TIM-3. This further demonstrates the dual effect of TIM-3 depending on ligand availability.

There are 4 known ligands to TIM-3, and Galectin-9 (Gal-9) was the first to be discovered. TIM-3-GAL9 interaction leads to selective apoptosis of CD4+ TH1 cells but not TH2 cells via intracellular calcium influx (181). GAL-9 expressed on macrophages also show a bidirectional effect, whereby TIM-3 binding activates intracellular pathways on macrophages to circumvent bacterial growth such as in tuberculosis (182) but dampens the partner T cell to control local tissue inflammation. Gal-9 is also expressed in multiple tumors (183), which impairs CD8+ cytotoxic killing as well as NK cell development and activation via reduction in IL-2 secretion (184). The ectodomain of TIM-3 also contains a specific cleft that binds to phosphatidylserine but does not interfere with Gal-9 binding as the two sites are separate. TIM-3 binding to phosphatidylserine results in the phagocytosis of apoptotic cells by APCs; however, similar engagement to phosphatidylserine with TIM-3 expressed on T lymphocytes does not lead to cellular clearance (185). Instead, this interaction may provide an apoptotic signal to the lymphocytes themselves. TIM-3 also binds to carcinoembryonic antigen cell adhesion molecule 1 (CEACAM1) which functions to fine-tune TIM-3 regulation. The expression of both TIM-3 and CEACAM-1 appears to be interdependent as reduction of one impairs the other. CEACAM1-TIM-3 binding also negates the costimulatory effects of CD3 and CD28 on T lymphocytes, while dual blockade leads to increased TIL frequency and tumor clearance in mouse models (186).

TIM-3 also affects several other immune cell subsets. TIM-3 overexpression augments effector function in Tregs leading to increased IL-10 secretion and T cell exhaustion (178). TIM-3 also marks NK cell maturation. While it does not appear to be in and of itself an exhaustion marker in NK cells, TIM-3 cross linking with antibody leads to suppressed NK cytotoxicity as well as decreased cytokine production (187). As described earlier, binding of TIM-3 on dendritic cells to HMGB1 leads to impaired endocytosis of PAMPs and nucleic acids from apoptotic tumor cells, which suppresses antigen presentation and the innate immune response (188). Finally, Gal-9-TIM-3 interaction has been observed to polarize TAMs towards an immunosuppressive phenotype, which was reversed with prolonged lipopolysaccharide stimulation exposure (189)).

The function of TIM-3 in GBM is still not fully elucidated. Unlike other ICs, TIM-3 is also expressed on tumor cells specifically in GBM (190). Its expression is strongly correlated with increasing glioma grade, IDH-wildtype, and mesenchymal subtype (191). It also seems to play a role in shaping the immune TME as certain chemokines involved in leukocyte migration and homing were correlated with higher TIM-3 expression in GBM (192). Interestingly, TIM3 expression specifically on primary microglia in the TME but not CD8+ TILs appears to be downregulated by GBM itself both *in vitro* and *in vivo*, resulting in impaired antigen specific activation of CD8+ T cells (193). However, this study used a variant of TIM-3 with a mutated cytoplasmic tail abrogating downstream intracellular signaling that was previously associated with augmenting T cell activation (194). GBM patients also exhibit higher TIM-3+ NK cells as well as anti-inflammatory M2-polarized macrophages compared to healthy controls (195). Finally, TIM-3 has direct pro-tumorigenic effects and contributes to overall tumor invasiveness, proliferation, migration and stemness in GBM through an IL-6 feedback loop via the NF-KB pathway activated through Gal-9-TIM-3 interaction (190).

Given the multiple modes of action across many immune cell populations and direct non-immunogenic effects, TIM-3 has become an appealing target for immunotherapeutic blockade. It has undergone early safety phase I trials in various solid tumors (NCT02817633) and has been studied in combination with other ICBs including PD-1 as well (NCT03680508 and NCT04139902). TIM-3 is not only much more significantly expressed in GBM compared to PD-L1 and CTLA-4 (190) but also correlated with a poorer prognosis and resistance to TMZ (196). These findings suggest that TIM-3 might play a more critical role in GBM immune escape. In a preclinical study investigating dual blockade of PD-1 and TIM-3 with or without focal radiation, triple therapy demonstrated 100% survival in mouse models compared to anti-TIM3 alone (197). Currently, there is a phase 1 trial investigating anti-TIM3 (MBG453) in combination with spartalizumab (anti-PD1) in combination with SRS in recurrent GBM patients, however no results have been published to date (NCT03961971). Further understanding of its mechanism of action, multiple ligand interactions, and synergistic effects on other ICs is clearly warranted.

## Discussion

The main ICB clinical trials in GBM have so far focused on replicating therapeutic success seen in other tumors through similar strategies including combining several ICBs with other immunotherapy modalities on top of the current standard of care. However, the highly immunosuppressive GBM TME significantly impairs the main immune cell players on which the efficacy of ICB therapy rests. Furthermore, the highly redundant and interconnected signaling pathways that underlie multiple ICs may render individual blocking antibodies ineffective. Understanding theses mechanistic pathways and their interplay with the immune landscape is therefore a critical step in advancing ICB therapy in GBM.

**Figure 3:**
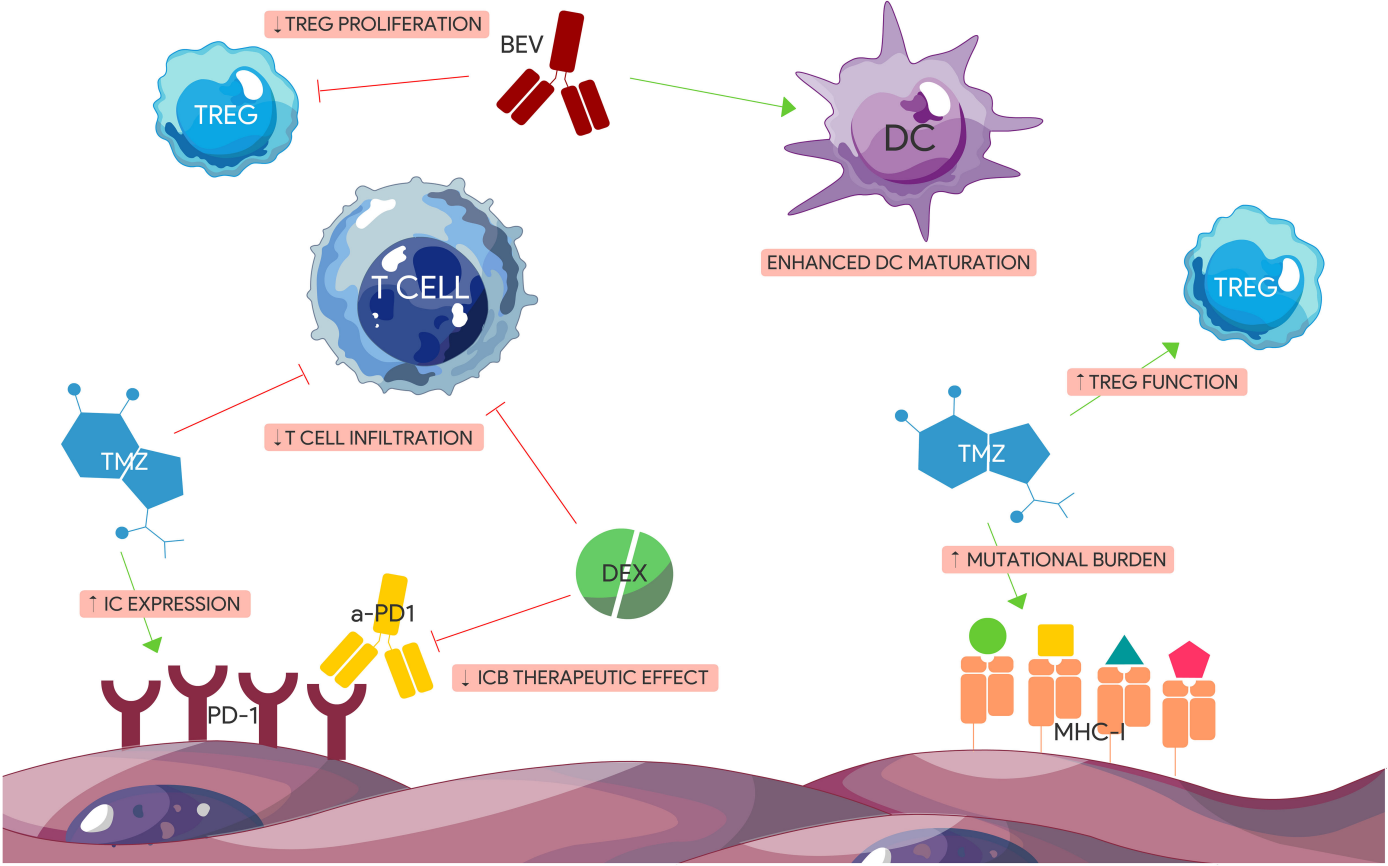
Immunogenic effects of standard glioblastoma treatment modalities. TMZ and Dex have immunosuppressive effects on GBM TME. Dex impairs the effect of anti-PD-1 therapy and reduces T cell tumor infiltration. TMZ induces the expression of certain ICs including PD-1, increases tumor mutational burden contributing to tumor heterogeneity, and augments TREG immunosuppressive function. BEV has pro-inflammatory effects including enhancing DC maturation and impairing TREGs. (BEV) Bevacizumab. (DEX) Dexamethasone. (IC) Immune checkpoint. (ICB) Immune checkpoint blocker. (TMZ) Temozolomide.

Most of the clinical trials involving ICBs are generally combined with standard therapy. However, the effects of temozolomide (TMZ), radiation, bevacizumab (BEV) and steroids on the TME can also be immunosuppressive (**Figure 3**). For example, TMZ has been demonstrated to induce lymphopenia (198), Treg expansion (199), and IC expression through activation of the JAK/STAT pathway (200). Combining an ICB with the standard Stupp protocol may therefore be counterintuitive. In contrast, a slightly modified continuous low dose of TMZ termed metronomic dosing (MD) has been shown to induce lower levels of immune cell depletion, IC expression particularly TIGIT and LAG-3 (201), and Treg activation (202) without sacrificing therapeutic efficacy (203). Furthermore, strong evidence shows that while TMZ exposure leads to the accumulation of mutations in GBM (204) and therefore a higher expression of neoantigens that could induce an augmented immune response, a high mutational load in recurrent GBM appears to comprise only a small population (205). In addition, specific mutations in DNA repair machinery such as DNA mismatch repair proteins (MMR) are strongly associated with prior TMZ treatment (206). While defects in MMR proteins lead to TMZ resistance and tumorigenesis, MMR deficiency has also been associated with better response to ICBs at least in NSCLC (207) and colorectal carcinoma (208). Interestingly, GBMs resulting from bi-allelic MMR deficiency syndrome (bMMRD), a highly penetrant condition characterized by germline mutations in any of the 4 MMR genes, harbor a significantly high mutational burden comparable to that of other solid tumors that respond well to ICBs (209). Nonetheless, a therapeutic advantage combining TMZ and ICBs over monotherapy alone can be seen in preclinical GBM models. While TMZ was also found to blunt the frequency of TILs compared to anti-PD-1 treatment alone (210), it appears that TMZ induced lymphopenia is reversible after drug discontinuation (211). Theoretically, concomitant administration of ICBs during TMZ treatment when the T cell compartment is dampened may not be ideal.

The effects of TMZ on the TME goes beyond direct cytotoxicity and can also have immunogenic effects, although the mechanisms are not fully elucidated. Further studies investigating the role of TMZ in combination with ICBs including optimal dosage and timing can therefore shed light on treatment resistance mechanisms as well as opportunities for optimization and synergy.

The use of steroids to manage symptoms secondary to edema also poses several challenges to ICB therapy. Like TMZ, steroids can cause profound lymphopenia through several mechanisms as well as a decrease in effector cell function (212). More importantly, steroids have been shown to negatively affect overall survival in GBM patients especially at higher doses (213, 214). This deleterious effect was also replicated in several GBM mouse models where the addition of steroids has shown to diminish the survival benefit of anti-PD-1 therapy in a dose-dependent manner (215, 216). Dexamethasone was also shown to attenuate costimulatory pathways for T cell activation and differentiation, leading to increased CTLA-4 expression and cell cycle impairment (217). This was reversed with CTLA-4 blockade, which rescued T cell differentiation. Understanding the myelosuppressive effects of steroids and their impact on ICB efficacy is critical especially given their widespread use for symptomatic treatment. Other anti-inflammatory agents such as receptor for advanced glycation end product (RAGE) inhibitors are currently under investigation as a viable alternative to steroid use (218), which could avoid further immunosuppression in GBM.

Radiotherapy has also been a cornerstone of GBM treatment alongside TMZ and similarly, it has a variable immunogenic impact on the TME. While classically considered immunosuppressive due to its direct negative effect on leukocytes (219), the overall impact of RT is more variable depending on dosing. RT can have several opposing immunomodulatory effects including induction of both proinflammatory and immunosuppressive cytokines (i.e. IFN- γ, IL-1B, TGF-β, CSF-1) (220, 221), upregulation of MHC I (222), promotion of leukocyte invasion, expression of surface molecules (i.e. damage-associated molecular patterns) that aid in the endogenous antitumorigenic response (223), and enrichment of immunosuppressive cells including Tregs and MDSCs in the TME (224, 225). Moreover, the abscopal effect of RT whereby antitumor response is seen in a lesion away from the original site of radiation help illustrate the immunogenic effect of RT beyond direct cytotoxicity. Through the induction of neoantigens in dying tumor cells, RT leads to APC priming and the activation of naïve T cells that mature into effector cells and home to distant target lesions (226). In a bilateral GBM mouse model, it was demonstrated that local RT combined with anti-PD-L1 treatment caused decreased viability of the contralateral non-irradiated GBM lesion with resulting increased overall survival compared to RT or anti-PD-L1 monotherapy alone (227). This effect appeared to be dependent on the expression of a neoepitope (EGFRvIII) and the influx of macrophages and T cells in the non-irradiated side. This synergistic effect of RT with ICBs has been demonstrated in other pre-clinical GBM models as well and appears to be mediated by an antigen specific immune response (228, 229). It is possible that standard RT dosing (60Gy delivered in 30 fractions), which was used in the larger ICB trials (Checkmate 498, Checkmate 143, and Checkmate 548), may initially confer immunogenic advantages, but could eventually contribute towards an immunosuppressive TME due to its longer course compared to hypofractionation (230). Current studies are currently exploring the effect of hypofractionated RT in combination with ICBs in order to capitalize on the positive immunomodulatory effects of RT while minimizing unintended immunosuppression (NCT02866747 and NCT05423210).

The anti-angiogenic drug bevacizumab has been a mainstay secondary treatment in recurrent GBM for several years to address symptoms secondary to edema despite having no significant impact on overall survival in GBM (231) unlike in other solid tumors (220, 221). While bevacizumab has positive immunogenic effects including enhancing DC maturation (232), inhibiting Treg proliferation (233), and reduction of PD-L1 expression in certain immune cells (234), it can also lead to peripheral immunosuppression and exacerbate lymphopenia when combined with TMZ and radiation (235). Despite its pro-inflammatory effects, bevacizumab may not be enough to synergize with anti-PD1 treatment as both mechanisms are affecting the same IC axis and may not compensate for the paucity of effector TILs in the TME. This could explain the similar overall survival benefit observed in either bevacizumab or Nivolumab monotherapy in recent trials (NCT02337491; NCT03661723). Despite these clinical outcomes, the pro-inflammatory effects of bevacizumab can still be crucial in future combinatorial immunotherapies and a way to reduce steroid use (236).

The timing of ICB administration may also be a critical factor in influencing the GBM immune microenvironment. A clinical trial by the Ivy Foundation at Barrow investigated the role of neoadjuvant anti-PD-1 in patients with surgically resectable recurrent glioblastoma and demonstrated that patients receiving pembrolizumab pre- and post-surgery had longer survival compared to those receiving adjuvant therapy alone (13.7 months *vs*. 7.5 months respectively) (237). The neoadjuvant arm also showed phenotypic changes in specific immune cell subsets including downregulation of PD-1 and upregulation of CTLA-4 on CD4+ T cells, a decrease in the intermediate monocytic population, and upregulation of IFN-γ responsive genes. Moreover, a focal upregulation of PD-L1 was noted in the neoadjuvant arm indicating early activation of lymphocytes in this group prior to surgery. Ultimately, the downregulation of cell-cycle-related genes appears to account for the main survival benefit in the neoadjuvant group. A separate single arm trial assessing the immunogenic effects of neoadjuvant nivolumab (NCT02550249) (238) on the TME showed neoadjuvant exposure increased TCR clonal diversity and upregulation of immune-related transcripts including chemo-attractants. While Cloughesy and colleagues were not able to detect an increase in TCR diversity, they noted expansion of T cell clones in the neoadjuvant group. In either case, the results from both studies underpin the immunogenic impact of neoadjuvant ICB therapy including the conditioning of a T cell response prior to surgery. This could result in the priming of the immune microenvironment to elicit a greater anti-tumor response while combating immune cell exhaustion.

Finally, tumor vaccines in GBM, while promising in theory, have showed modest clinical survival benefit at best (NCT00643097 and NCT01280552) (239). Targeting tumor specific antigens (TSAs) in GBM does not account for the highly heterogenous nature of the tumor leading to eventual antigen escape of untargeted cells such as in the case of EGFRvIII (240). Moreover, the primed and activated effector T cells are then exposed to the same immunosuppressive TME resulting in exhaustion. The addition of ICBs to GBM tumor vaccines may allow for a more robust and continued immune response by preventing T cell dysfunction, improving T cell response, and augmenting the TIL population (241). Other preclinical studies also demonstrate a reduction in Tregs and expansion in both CD4+ and CD8+ effector subsets (242). In fact, this combinatorial therapy has seen some clinical success in other solid tumors (243, 244). In GBM, several preclinical studies have demonstrated therapeutic benefits of combinatorial therapy (245, 246). Liu and colleagues treated CT2A GBM mouse models with anti-PD-L1 and a polyvalent vaccine targeting 3 neoantigens and noted superior survival than either monotherapy alone. The CT2A GBM model was used as it is not as sensitive to ICB therapy compared to other models including GL261 and more closely mirrors the ICB response seen in human GBM. It is also important to note that CT2A harbors a higher tumor mutational burden compared to its human counterpart, which could play a significant role in boosting the synergistic effect of this regimen. Currently, there are several on-going clinical trials investigating the efficacy of tumor vaccine with ICBs in GBM (NBCT03018288, NCT03422094, NCT03750071).

## Conclusion

Although ICB therapy in GBM has not replicated promising results similar to other solid tumors, there are still opportunities to harness the immune response to combat this fatal disease. Understanding the molecular mechanisms that underlie ICs is critical in designing future combinatorial therapies that can optimally synergize and overcome redundant and compensatory pathways that lead to treatment failure. Furthermore, revisiting the standard of care treatments and their biological and immunogenic impact will be critical in moving ICB trials and GBM therapies in general forward.
